# Diagnostic, Prognostic, Predictive, and Monitoring Role of Neutrophil CD11b and Monocyte CD14 in Neonatal Sepsis

**DOI:** 10.1155/2021/4537760

**Published:** 2021-10-14

**Authors:** Heba E. Hashem, Zakaria H. Ibrahim, Wafaa O. Ahmed

**Affiliations:** ^1^Ain Shams University, Egypt; ^2^Al Azhar University, Egypt

## Abstract

**Background:**

Sepsis is a critical medical condition that requires additional diagnostic considerations. Recently, focus has shifted to the diagnosis of sepsis using new markers to overcome the limitations of traditional laboratory diagnostic modalities. Neutrophil CD11b (nCD11b) and monocyteCD14 (mCD14) cell surface antigens have been shown to be useful in such diagnostic consideration.

**Aim:**

To investigate the diagnostic, monitoring, prognostic, and predictive roles of nCD11b and mCD14 as sepsis biomarkers in comparison to each other and to traditional laboratory sepsis parameters in order to select the best fit for routine daily use in neonatal intensive care units (NICUs).

**Subject:**

The study included 188 neonates from Ain Shams University Hospitals' NICUs, who were divided into two groups: the control group (*n* = 100) and the sepsis group (*n* = 88). Highly sensitive CRP (hs-CRP), complete blood count (CBC), blood culture, and nCD11b and mCD14 evaluations were all part of the laboratory sepsis evaluation (done by flow cytometry technology). Positive blood culture results (BACT/ALERT system) confirmed the sepsis diagnosis. Twenty-four enrolled sepsis neonates were subjected to follow-up assessments, and they were divided into two groups based on clinical improvement: improved sepsis and sepsis without improvement. In order to predict performance evaluation, the subjected neonates were reclassified according to their outcome into survivors' and nonsurvivors' group.

**Results:**

Sepsis patients had a significant increase in mCD14 MFI values when compared to controls. With sensitivity 75.4 percent, specificity 71.9 percent, efficacy 73.3 percent, and AUC 0.703, mCD14 MFI at cutoff 9.36 could distinguish the presence of septicemia. Significant increases in both mCD14 MFI and nCD11b MFI (*P* = 0.001) were observed in the severe sepsis/septic shock group compared to the nonsevere sepsis group. The combined measurement of CD14 MFI at cutoff 9.97 and CD14 percent at cutoff 44.7 percent yielded the best predictive performance.

**Conclusion:**

Sepsis patients had a significant increase in mCD14 MFI comparable to the controls. mCD14 MFI demonstrated better diagnostic, prognostic, and predictive results than nCD11b. hs-CRP outperformed mCD14 and nCD11b in terms of diagnostic efficacy and AUC. In the monitoring of sepsis patients, both mCD14 and nCD11b produced unsatisfactory results. Currently, the routine use of mCD14 or nCD11b as sepsis biomarkers in neonatal ICUs is not justified.

## 1. Introduction

Sepsis in newborns is a common critical medical situation associated with high mortality rates as well as many neurodevelopmental disabilities among survivors; thus, early detection and management of neonatal sepsis (NS) is critical [1, 2].

The blood culture is the gold standard for sepsis diagnosis; however, its results are usually delayed for days [3], and it has unsatisfactory diagnostic performance in some circumstances, particularly in neonatal ICUs where the inoculated blood volume is usually critical. As a result, broad-spectrum antibiotics are usually given to all suspected neonates in order to prevent potentially serious complications [4, 5]. This empiric antibiotic therapy resulted in unnecessary antibiotic exposure, as well as the emergence of drug-resistant strains and the high neonatal healthcare costs spent each year [6].

Because current laboratory diagnostic tools have many limitations and confounding factors, a challenge between a large battery of sepsis biomarkers was established in order to find the ideal diagnostic and prognostic parameter that can overcome these conventional diagnostic obstacles [7]. Neutrophil CD11b (nCD11b) is a current researchable cell surface marker that is thought to be useful in the diagnosis of sepsis. It functions as an Fc-receptor, which is found on the surface of nonactivated neutrophils but in low concentration, and can be significantly upregulated when the sepsis process begins [8–10].

Neutrophil CD11b (nCD11b) is one of the current researchable cell surface markers that is thought to be useful in the diagnosis of sepsis. It functions as an Fc-receptor, which is found on the surface of nonactivated neutrophils but in low concentration, and can be significantly upregulated with the onset of the sepsis process [11]. It has been reported to be a highly effective marker in the diagnosis of early-onset sepsis, with higher expression in infected neonates than in noninfected neonates [12].

Monocyte CD14 (mCD14) is a glycoprotein cell surface marker found on the surface membranes of monocytes and macrophages that functions as a receptor for complexes of lipopolysaccharide (LPS) and LPS-binding proteins. It promotes the inflammatory response of the host by activating a specific proinflammatory signalling cascade against the various infectious agents [13]. It has been implemented as a valuable diagnostic biomarker of septicemia [14].

Previous research suggests that both nCD11b and mCD14 play important roles in patients with sepsis, but few studies have compared the diagnostic and monitoring values of both biomarkers combined in the same sepsis attack, so our study is aimed at investigating and comparing the clinical use of nCD11b and mCD14 as diagnostic, prognostic, monitoring, and predictive biomarkers (NICUs).

## 2. Materials and Methods

### 2.1. Study Design

The present study is a case-control prospective study carried out during the period from September 2018 till June 2020, and subjected neonates were selected from two neonatal intensive care units (NICUs) (NICU of Obstetric and Gynecological Hospital-Ain Shams University and NICU of Pediatric Hospital-Ain Shams University, Cairo, Egypt). All procedures were following the Helsinki Declaration [15]. Informed written consent was received from the parents of the subjected neonates, and the study was approved by the Research Ethics Committee of Ain Shams University Hospitals, Faculty of Medicine.

### 2.2. Sepsis Identification

Neonates were recruited in the study according to the New Ballard score [16]. For sepsis patient's identification and selection, neonates had presumed one or more infection risk factors [17], in addition to at least 2 clinical and 2 laboratory criteria which are described in detail in previous studies [18–20]: (1) respiratory rate > 60 breaths per minute or cessation of respiration for ≥20 seconds, occurring at a rate of ≥2 times per hour, or pulse oximeter readings of ≤85%; (2) heart rate of <100 beats per minute, pallor, or hypotension; (3) hypothermia (rectal temperature of <36°C), a body temperature of >38°C, feeding intolerance (increased gastric residuals of >50% of milk volume in ≥2 feedings within 24 hours), glucose instability (blood glucose level of <45 mg/dL or >125 mg/dL), or metabolic acidosis (pH < 7.25); or (4) lethargy or decreased activity, whereas laboratory criteria were white blood cell (WBC) count <5 or >20 × 10^9^ cells/L, immature to total neutrophil (I : T) ratio > 0.2, platelet count < 100 × 10^9^/L, and CRP > 10 mg/L.

#### 2.2.1. Exclusion Criteria

Patients who had recently undergone surgical interventions (within the last 15 days) were excluded from the study to avoid the effect of the surgical process on the levels of the studied inflammatory biomarkers, as were patients who had been admitted for more than one month and neonates who had confirmed intrauterine viral infection (toxoplasmosis, rubella, and Cytomegalovirus).

The present study represents a part of our group of researches being concerned about evaluating the diagnostic and prognostic performance of cell surface markers as sepsis indicators. Group classification, patient evaluation, and laboratory investigations in the current study were designed in concordance with these previous researches [20, 21].

### 2.3. Group Classification

The neonates were divided into two groups: the control group and the sepsis group. The controls (group 1) were postnatal age- and sex-matched newborns who showed no signs of infection (sepsis inclusion criteria were excluded in addition to negative hs-CRP results).

The controls were subdivided into two subgroups: (group 1a) healthy controls, which included healthy neonates (cord blood samples), and (group 1b) diseased control group, which included those neonates with no signs of infection but subjected to sampling for performing investigations of different diseases including neonatal physiological jaundice, neonatal hypoglycemia, neonatal convulsions, Hirschsprung disease, and duodenal atresia, infants of diabetic mothers (IDM), and premature neonates.

The identified sepsis neonates (group 2) were further subdivided into two subgroups: (group 2a) documented sepsis patients, which included those neonates with the clinical diagnosis of sepsis plus positive blood culture, and (group 2b) clinical sepsis patients, including those neonates with clinical diagnosis of sepsis but with negative blood culture results.

In the case of sepsis patients, they were further classified based on the severity of the disease into two groups: those with severe sepsis/septic shock, who met the clinical criteria for disease severity, and those with nonsevere sepsis.

The severity of neonatal sepsis was determined by the presence of sepsis plus one of the following: cardiovascular organ dysfunction, acute respiratory distress syndrome, or two or more other organ dysfunctions. Septic shock is the next stage of the sepsis continuum, which occurs when multiple inotropes are used without clinical benefit, followed by neonatal death [22].

In addition to diagnostic and prognostic sepsis evaluations, enrolled neonates underwent follow-up assessments. On the fifth day following the baseline evaluation, the second clinical and laboratory evaluations were performed. The neonates were divided into two groups based on their clinical condition (group 1: nonimproved sepsis patients and group 2: improved sepsis patients).

Additionally, for predictive performance evaluation, the subjected patients were reclassified according to their outcome into survivors' group and nonsurvivors' group.

### 2.4. Patient Evaluation and Laboratory Investigations

In the current study, newborns were given a detailed medical history and a thorough clinical examination, as well as peripheral blood sampling for laboratory sepsis investigations. All laboratory investigations were performed at the Clinical Pathology Departments of Ain Shams University Hospitals. Blood samples from the sepsis group were withdrawn as soon as the neonate was clinically suspected to have sepsis signs and symptoms and before the initiation of antibiotic therapy.

The laboratory sepsis profile included the following: complete blood count (CBC) and peripheral blood smears for the differential count, highly sensitive CRP (hs-CRP) using Flex reagent cartilage, and chemistry profile for both clinical judgment and follow-up purposes including alanine transaminase (ALT), aspartate transaminase (AST), blood urea, serum creatinine, and arterial blood gases.

Blood culture was used to confirm sepsis diagnosis: 2 mL of blood was injected into the blood culture bottle under complete aseptic conditions. The inoculated culture bottles were examined daily. Positive samples were Gram-stained and subcultured on blood agar, MacConkey agar, and chocolate agar; then, plates were incubated at appropriate temperatures. Full identification of organisms was done following the standard operating procedures (SOPs) of our microbiology unit.

Surface nCD11b was measured by using the Leuko 11b assay (Leuko11b kit, Trillium Diagnostics, Scarborough, ME, USA), and FITC-labeled mouse anti-human CD11b was used (Becton Drive BD Biosciences product). Data analyses were performed using a Becton-Dickinson FACScan system.

For sample preparation, peripheral blood samples on EDTA were processed and analyzed within two hours, up to 48 hours maximum of sample collection time. Briefly, 50 *μ*L of well-mixed anticoagulated whole blood was incubated for ten minutes at room temperature with saturating amounts of fluorescein isothiocyanate-conjugated anti-CD11b murine monoclonal antibody or isotype control (Leuko11b kit; Trillium Diagnostics), followed by ammonium chloride-based red cell lysis.

Samples were washed once and resuspended in 0.5 mL of phosphate-buffered saline with 0.1% bovine serum albumin. Flow cytometric analyses were performed using a Becton-Dickinson FACScan system to collect data on the logarithm of green fluorescein isothiocyanate, and a linear right-angle side and forward scatter for a minimum of 5000 events were studied.

Surface monocyte CD14 flow cytometric analysis was conducted (Becton, Dickinson, and Company, USA; Accuri™ C6 Cytometer, USA), and it is performed by using the Mono 14 assay (Mono 14 kit, Trillium Diagnostics, Scarborough, ME, USA). FITC-labeled mouse anti-human CD14 was used (BD Pharmingen, BD Biosciences, USA), and sample processing was similar to nCD11b procedure steps.

#### 2.4.1. Gating Strategy

Using CD45 and side scatter (CD45/SS), initial gating was performed on the neutrophil area in the dot plot graph with nCD11b while gating was done on the monocyte area in the dot plot graph for mCD14 results. Data were expressed as mean fluorescence intensity (MFI) and percentage (%) for each marker using a single histogram, and each of the expression units for the two biomarkers was statistically analyzed.

### 2.5. Statistical Analysis

Statistical analysis was performed by using the SPSS statistical software package (IBM SPSS statistics V. 24.0). Data were expressed as number (percentage) for presenting qualitative data and median (interquartile range) for quantitative nonparametric data. The comparison between every two independent groups was done by the Wilcoxon rank sum test, in addition to the correlation statistics (Spearman correlation) for the possible associations between every two studied variables. The receiver operating characteristic (ROC) curve was used to assess the best cutoff point with sensitivity, specificity, positive predictive value (PPV), negative predictive value (NPV), and area under the curve (AUC) being calculated. The significance level was taken at *P* value ≤ 0.05 (*P* value at 0.05 or less was considered significant, while *P* values at 0.01 and 0.001 or less were highly significant). Additionally, for follow-up and monitoring purposes, delta change (dC) percentage for each biomarker was calculated and compared.

## 3. Results

In the current study, 188 neonates were recruited, 84 males and 104 females, with a male to female ratio of 1 : 1.23. They were divided into two groups: control (*n* = 100) and sepsis (*n* = 88). The control group was further subdivided into (group 1a, *n* = 52) healthy controls and (group 1b, *n* = 48) diseased controls, while patients with sepsis were further subdivided into (group 2a, *n* = 40) patients with documented sepsis and (group 2b, *n* = 48) patients with clinical sepsis. The comparison was made between sepsis neonates and controls. Because the data were nonparametric, the Wilcoxon rank sum test was used. The demographic and clinical data of the groups studied are depicted in Table 1.

### 3.1. Blood Culture Results

**Figure 1:**
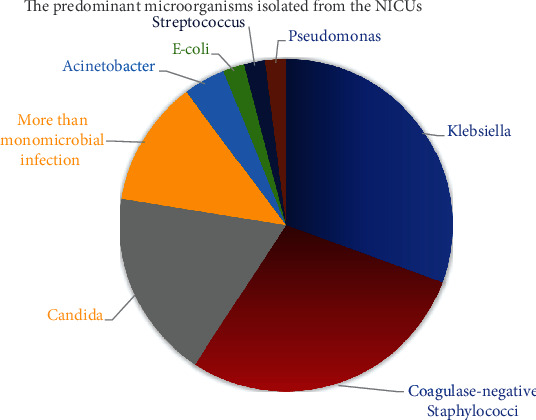
The distribution of the causative microorganisms.

Blood cultures were positive in 55.6% of all neonates with sepsis. *Klebsiella* was the predominant microorganism isolated from NICU patients with sepsis (*n* = 15, 17%), followed by coagulase-negative *Staphylococci* CONS (*n* = 14, 15.9%) and *Candida* spp. (*n* = 9, 10.2%). Other results are less commonly encountered in our NICUs including more than monomicrobial infection (*n* = 6, 6.8%), *Acinetobacter* (*n* = 2, 2.27%), *E. coli* (*n* = 1, 0.88%), *Streptococcus* spp. (*n* = 1, 0.88%), and *Pseudomonas* spp. (*n* = 1, 0.88%) (Figure 1).

### 3.2. Outcome of Sepsis Patients

There was a highly significant increase in the mortality rate among patients compared to the controls (*P* < 0.001). We investigated the outcome of sepsis until discharge from NICU; 28/88 (31.8%) died from severe septicemia and its complications, while 57/88 (64.7%) showed clinical improvements and discharge. 3/88 (3.4%) were transferred to other NICUs (on parents' demand and/or to perform required surgical interventions).

#### 3.3. Laboratory Evaluation

**Figure 2:**
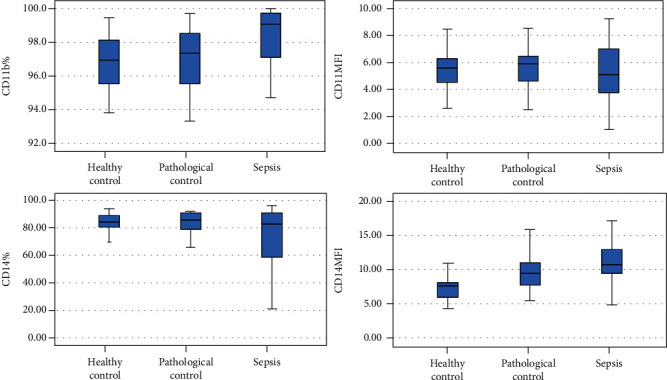
nCD11b%, nCD11b MFI, mCD14%, and mCD14 MFI box blots for the healthy controls, diseased controls, and group of patients with sepsis. nCD11b%: no significant difference in sepsis neonates (median (IQR): 99.05 (97.1-99.7)%) when compared to either the healthy controls (median (IQR): 96.95 (95.525-98.1)%) and the diseased controls (median (IQR): 97.35 (95.5-98.5)%); additionally, no significant difference was found upon comparing healthy and diseased controls together (*P* = 0.435). nCD11b MFI: no significant difference was documented when the sepsis group (median (IQR): 5.09 (3.7525-7)) was compared to both the healthy controls (median (IQR): 5.555 (4.4575- 6.22)) and the diseased controls (median (IQR): 5.905 (4.625-6.4525)), and no significant difference was found upon comparing healthy and diseased controls (*P* = 0.283). mCD14%: no significant difference was documented when the sepsis group (median (IQR): 82.7 (58.88-90.7)%) was compared to both the healthy controls (median (IQR): 84.15 (80.075-89.05)%) and the diseased controls (median (IQR): 85.7 (79.1-90.9)%), and no significant difference was found upon comparing healthy and diseased controls (*P* = 0.291). mCD14 MFI: it was significantly elevated (*P* < 0.001) in the sepsis group (median (IQR): 10.8 (9.315-13.2)) when compared to the healthy controls (median (IQR): 7.8 (6-8.23)), while no significant difference was reported (*P* = 0.157) when the diseased controls (median (IQR): 10.015 (7.7725-11.2)) were compared with the sepsis group. Also, a statistically significant difference was found when healthy and pathological controls were compared together (*P* ≤ 0.001) being higher in the diseased controls.

Laboratory parameters were compared among healthy controls, diseased controls, and patients with sepsis and illustrated in Table 2 and Figure 2.

Additional statistical comparison between the controls and documented sepsis patients was performed, and it was evident that nCD11b MFI, hs-CRP, PLT, Hb, and ALC showed significant differences between both groups (Table S1).

Table 3 illustrates the comparative statistical analysis between documented and clinical sepsis as regards the laboratory parameters. hs-CRP, nCD11b MFI, mCD14 MFI, Hb, PLT, TLC, AMC, and ALC showed significant differences between both subgroups.

Sepsis patients were furtherly regrouped according to the severity of the disease into severe sepsis/septic shock group (*n* = 22) and nonsevere sepsis patients (*n* = 66), the comparison was conducted between both groups, and the results are illustrated (Table 4).

The diagnostic utility for each marker and their combinations are shown in Table 5 and Figure 1).

**Figure 3:**
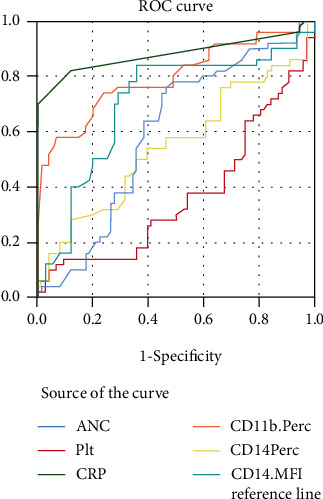
ROC curve shows the diagnostic role of the studied biomarkers for the differentiation between patients with sepsis and the control groups.

The ROC curve for nCD11b%, mCD14%, mCD14 MFI, CBC parameters, and hs-CRP is illustrated in Figure 3, and the AUCs of the studied sepsis parameters were calculated (Table 6).

Note that the figure was drawn for only those having the best cutoff, and there were no representative lines for the following parameters: Hb, TLC, ALC, AMC, and nCD11b MFI, as they did not satisfy both specificity and sensitivity > 50%.

The multiregression analysis was conducted to find the best panel of markers that can discriminate effectively between patients with sepsis and the controls (Table 7). The first panel included all the performed sepsis parameters, and *P* values were calculated for each; accordingly, the biomarker that gave a significant value was included for further analysis. The final regression model enrolled both nCD11b% and hs-CRP which achieved the highest *F*-ratio that could be concluded from the present study.

Concerning correlation statistics, both nCD11b and mCD14 failed to show a significant correlation with the other studied parameters except for a positive correlation between both biomarkers of MFI (i.e., nCD11b MFI and mCD14 MFI) (*r* = 0.299, *P* = 0.024). A nonsignificant correlation was documented between hs-CRP and any of nCD11b% (rs = −0.027, *P* = 0.811), nCD11b MFI (rs = −0.187, *P* = 0.099), mCD14% (rs = 0.056, *P* = 0.627), and mCD14 MFI (rs = −0.187, *P* = 0.176) (Table S2).

### 3.4. Monitoring Performance of Analyzed Sepsis Biomarkers

After 5 days from the baseline evaluation, twenty-four of the recruited neonates had a follow-up assessment. Clinically, they were divided into two groups: group 1 (patients with improved sepsis) (*n* = 15) and group 2 (patients with nonimproved sepsis) (*n* = 9).

The comparative analysis was conducted between the 1^st^ and the 2^nd^ monitoring evaluations for each patient in both groups. The group of patients with improved sepsis showed significantly different values between both initial and follow-up evaluations regarding hs-CRP, PLT, nCD11b MFI, ANC, and Hb (Table S3), while for nonimproved sepsis patients, all measured sepsis markers showed nonsignificant differences (>0.05) between both evaluations concluding their usefulness in the follow-up of nonimproved sepsis (Table S4).

### 3.5. Delta Change % Statistical Evaluation

Delta change (dC) % for each biomarker was calculated, and the statistical calculation method is described in detail in other studies [20, 21]. dC *Z* values for nCD11b%, nCD11b MFI, mCD14%, and mCD14 MFI were -0.567, -0.397, -1.756, and - 0.736, respectively (Table 8). These findings highlight the unsatisfactory role of both nCD11b and mCD14 in the follow-up and monitoring of neonates with sepsis for both neonates with improved and nonimproved sepsis, simultaneously.

### 3.6. Predictive Performance of Sepsis Biomarkers for 28-Day Mortality

After analyzing the diagnostic, prognostic, and monitoring performance, the predictive performance of the sepsis markers nCD11b and mCD14 was evaluated (i.e., predicting the patient's mortality based on his preliminary test evaluation result). The valuable predictive capability can greatly assist clinicians in tailoring their appropriate management protocol from the start and allowing them to consider which patients have a potential risk and thus require careful observation. The subjected patients were reclassified according to their outcome into survivors' group (*n* = 60) and nonsurvivors' group (*n* = 28). The predictive validity tests for each parameter were calculated, and the comparison between each of them was conducted (Table 9).

The results illustrated that mCD14 MFI could achieve the most desirable univariable predictive value with sensitivity 66.7%, specificity 70.3%, efficacy 69.2%, and AUC 0.846, followed by mCD14% achieving sensitivity 54.5%, specificity 72.9%, efficacy 67.1%, and AUC 0.594, while the remaining sepsis parameters achieved lower predictive performance. Multi-ROC curve analysis showed that the highest predictive performance could be registered by a combined measurement of CD14 MFI at cutoff 9.97 and CD14% at cutoff 44.7% achieving sensitivity 66.7%, specificity 97.3%, efficacy 88.5%, and AUC 0.673, respectively.

## 4. Discussion

Neonatal sepsis is a potentially fatal medical condition that affects developing countries, including Egypt. It is linked to a high number of fatalities as well as lifetime morbidities and disabilities among survivors [23]. A battery of sepsis biomarkers have recently been investigated to determine which is the ideal marker for routine daily use and overcoming diagnostic challenges encountered with current conventional laboratory diagnostic modalities. The cell surface markers are at the top of the list of these newly studied biomarkers; the clinical utility of both neutrophil CD11b (nCD11b) and monocyteCD14 (mCD14) as sepsis biomarkers has been demonstrated to be beneficial in this regard [11, 12, 14].

In the current study, neonates with clinical sepsis (*n* = 88) were recruited, while a control group of 100 neonates with no signs or symptoms of sepsis was included. The number of deaths in the sepsis group was 28/88 (31.8 percent), reflecting the high morbidity and mortality rates associated with fulminant septicemia, as supported by other studies [24–26].

In terms of blood culture results, it was discovered that 55.6 percent of all septic neonates had positive blood cultures, with *Klebsiella* being the most common microorganism isolated from NICU sepsis patients (17%), followed by coagulase-negative *Staphylococci* CONS (15.9%). These findings are consistent with other Egyptian studies that found *Klebsiella* spp. and coagulase-negative *Staphylococci* CONS to be the most common organisms isolated from sepsis neonates [26, 27].

Regarding the diagnostic performance of the studied sepsis biomarkers, nCD11b% showed no significant difference in sepsis patients comparable to both healthy controls and diseased controls. Additionally, no significant difference was found between healthy and diseased controls when compared together (*P* = 0.435). nCD11b% at cutoff 99% achieved specificity 94.0%, sensitivity 50.0%, PPV 87.2%, NPV 69.6%, efficacy 74.2%, and AUC 0.798.

mCD14 MFI significantly increased (*P* < 0.001) in sepsis when compared to that in the healthy controls, but no significant difference was reported (*P* = 0.157) when the diseased controls were compared with the sepsis patients. Additionally, a statistically significant difference of CD14 values exists when healthy and diseased controls were compared together (*P* ≤ 0.001) being higher in the diseased controls than in the healthy controls. mCD14 MFI at cutoff 9.36 could discriminate the presence of septicemia with sensitivity 75.4%, specificity 71.9%, PPV 63.2%, NPV 82.1%, efficacy 73.3%, and AUC 0.703, respectively.

These suboptimum diagnostic validity results of both nCD11b and mCD14 were agreed upon by some studies [28–31] while rejected by others [12, 32]. Fitrolaki et al. [30] evaluated the diagnostic validity of nCD11b in neonatal septicemia, and they documented that there was no significant difference in its levels between sepsis neonates and healthy controls.

Recently, ELMeneza et al. [33] concluded that CD11b is a sensitive marker for sepsis in full-term neonates that can be introduced into routine daily work because the percentage of neutrophils expressing CD11b was significantly upregulated in the sepsis and suspected sepsis groups versus the controls. The main limitation of their study is that they used clinical sepsis scores, haematological pictures, CRP, and ESR to confirm the diagnosis rather than microbiological cultures, which could explain the disparity between their results and the current study results.

Turunen et al. [29] added that the role of CD11b in neonatal infections is still debatable, explaining his findings by its widespread sensitivity and specificity between studies that were affected by other conditions such as respiratory distress syndrome.

mCD14 was evaluated as a marker of sepsis severity in addition to its diagnostic value. Patients with severe sepsis/septic shock were compared to those with nonsevere sepsis. There was a significant difference in mCD14 MFI values (*P* = 0.001), which were lower in severe sepsis cases. Furthermore, mCD14 MFI shows a significant difference (<0.001) between documented sepsis (positive blood culture) and query sepsis (negative blood culture) patients, indicating that mCD14 MFI may have a valuable prognostic role in addition to its role in sepsis diagnosis. These results came concordant with Schaaf et al. [34] who reported increased expression of CD14 on monocytes of patients with sepsis as compared to controls. Also, increased mortality is associated with downregulation of CD14 expression on monocytes.

Concordant with our study results, Jia et al. [35] demonstrated that the surface expression of HLA-DR/CD14 on peripheral blood mononuclear cells is closely related to the severity of infection and can be used to differentiate the severity of critical illness in ICU settings.

The predictive performance of the studied sepsis markers was also assessed. The predictive statistical analysis was aimed at determining which of the studied biomarkers could accurately predict end-organ dysfunction before it was too late for intervention, resulting in improved patient survival. This evaluation is critical for triaging patients with sepsis because it has been discovered that delaying ICU admission can worsen the condition [36]. For the predictive assessment, the patients were divided into two groups based on their outcome: survivors (*n* = 60) and nonsurvivors (*n* = 28). The findings revealed that mCD14 MFI and mCD14 percent were the most biomarkers capable of predicting 28-day mortality in sepsis patients. These findings are supported by Aalto et al. [37] who found that low monocyte mCD14 expression predicts 28-day mortality in sepsis patients.

On the other hand, highly sensitive CRP, which is the standard sepsis biomarker used in our NICUs, has become available in various clinical laboratories. Furthermore, this ultrasensitive immunoturbidimetric and nephelometric assay can detect CRP at concentrations as low as 0.02 mg/dL [38]. In the current study, hs-CRP levels in sepsis were significantly higher (*P* < 0.001) than those in both healthy and diseased controls. Furthermore, it outperformed nCD11b in terms of diagnostic performance and outperformed mCD14 in terms of specificity, PPV, efficacy, and AUC. At a cutoff value of 6 mg/L, hs-CRP had sensitivity 70.2%, specificity 94%, NPV 79.0%, PPV 90.8%, efficacy 83.2%, and AUC 0.880. These mentioned results suggest that the implementation of either of the studied cell surface markers, nCD11b or mCD14, as sole diagnostic markers in the routine clinical application is not advised.

Moreover, the multiregression analysis was in line with the multi-ROC curve study, the highest *F*-ratio was achieved by combined measurement of nCD11b% and hs-CRP, and these results are confirmed by other studies who reported that combined measurement of nCD11b% and hs-CRP could add a value to the diagnostic performance of nCD11b [12, 33]. Genel et al. [12] were in line with our results as they reported that a combination of CD64, CD11b, and CRP increases the sensitivity of the expression and the negative prediction of sepsis. There is no doubt that combination of these different biomarkers can enhance their sensitivities and/or specificities, due to the dynamic complexity of sepsis [39].

On the other side and comparable to the results previously reported from the multiregression analysis for other studied sepsis biomarkers which are documented by our previous studies, the reported *F*-ratio achieved by combined measurement of nCD64 and hs-CRP was 52.206 [21] while nCD64% and presepsin combined measurement achieved *F*-ratio 226.065 [20] which exceeds that is documented in the present study by hs-CRP and nCD11b% *F*-ratio (22.4) pointing to the unsatisfactory result of CD11b comparable to both CD64 and presepsin.

The correlation statistics of the current study revealed that both nCD11b and mCD14 failed to show a significant correlation with the other studied sepsis parameters including hs-CRP, and these results are supported by other studies' results [30, 33, 40].

The monitoring capability of each of the studied sepsis parameters was evaluated in addition to their diagnostic, prognostic, and predictive performance. Twenty-four of the recruited neonates had an additional follow-up after 5 days from the baseline evaluation; those neonates were chosen from the 88 neonates as the only ones who completed the follow-up evaluation period; the rest were discharged, referred, or died before the second-time evaluation. Clinically, they were divided into two groups: improved and nonimproved sepsis. The delta change results reflect the unsatisfactory performance of both nCD11b and mCD14 in monitoring sepsis patients.

By comparing the diagnostic and monitoring role of either nCD11b or mCD14 with that achieved previously by either of nCD64 or presepsin, being documented by other studies [20, 21, 41], it is evident that nCD11b and mCD14 are less ideal to be used as sepsis biomarkers in NICUs.

From a technical point of view, both nCD11b and mCD14 measurements were easy as flow cytometric measurement is rapid (turnaround time ends in a maximum of 2 hours), and they need only a small blood volume to be performed (50 𝜇L of whole blood is sufficient), in addition to their cost which is considered to be reasonable (60 L E/test). Concerning CRP, it is available in the laboratories with a lower financial cost comparable to these new biomarkers, reasonable turnaround, and high specificity [42]. In addition, CRP levels fall more rapidly with effective treatment. On the other hand, the rise of CRP correlates poorly with the course of the disease and interpretation can be affected by other physiological and pathological factors which make its use as a sole sepsis marker is not advised [38, 40].

Finally, despite the technical ease of routine application of either nCD11b and mCD14, moreover the median values of mCD14 MFI were significantly higher among sepsis patients than the controls (*P* < 0.05), but both nCD11b and mCD14 failed to achieve desirable results being lower than hs-CRP in their diagnostic validity results in addition to their unsatisfactory results in sepsis monitoring evaluations and lower statistical performance than other sepsis biomarkers being reported by the previous literature; this by its turn makes them less ideal to be clinically applied in NICU routine daily work.

## 5. Limitations of the Study

The main limitations of this study must be documented, (1) neonates with congenital anomalies, chromosomal abnormalities, and surgical procedures were not excluded from the study; besides, the control group included nonsepsis diseased neonates as well as healthy controls. Indeed, this was intended to test the clinical application of sepsis biomarkers in the different heterogeneous groups of patients which reflect the daily struggles of neonatal ICUs. (2) Many parameters of the demographic data showed statistically significant difference between both control and septic neonates, and this could affect the results of this research; however, indeed, it is suspected that sepsis patients have many risk factors and differences in the demographic data than the controls; actually, this is a reflection of NICU daily circumstances. (3) For follow-up purposes, only twenty-four neonates were chosen out of the 88 neonates as those only who completed the follow-up evaluation period, and the remainder either discharged, referred, or died before the complete evaluation.

## 6. Conclusion and Recommendation

Significant increase in mCD14 MFI values in sepsis patients compared to healthy controls was achieved comparable to CD14%, CD11b%, and CD11b MFI. Lower diagnostic validity results of either CD14 or CD11b were documented being lower efficacy than hs-CRP. Any of nCD11b, mCD14, and hs-CRP cannot be used individually as sole sepsis markers in sepsis diagnostic purposes. The best diagnostic test for early sepsis diagnosis can be documented by the current study which is reached by combined measurement of both nCD11b% and hs-CRP.

Both CD11b and CD14 are associated with unsatisfactory results in follow-up and monitoring evaluations of sepsis patients. mCD14 MFI achieved better results over CD11b in reflecting sepsis severity, sepsis patient's prognostic determination, and the prediction for 28-day mortality. One should interpret the results of any of the sepsis biomarkers in correlation with the patient medical condition for the best clinical judgment.

The high rate of neonatal mortality results from neonatal septicemia and its complications; moreover, the expected lifetime disabilities among the survivors necessitate the use of early diagnostic, best prognostic, and sensitive monitoring sepsis markers. Further studies are recommended to investigate the diagnostic, prognostic, monitoring, and prediction role of either nCD11b% and mCD14 MFI comparable to other valuable sepsis markers and with more serial follow-up evaluation episodes of sepsis patients. Up to date and based on the present study results, the routine application of any of nCD11b and mCD14 as a part of the sepsis evaluation profile in NICUs is not justified.

## Figures and Tables

**Table 1:** Demographic and clinical data of the studied neonates.

Parameter	Healthy control group (*n* = 48)	Diseased control group (*n* = 52)	Sepsis group (*n* = 88)
Preterm (GA < 37 w)	12 (25%)	17 (32.6%)	46 (52.27%)
LBW and VLBW	12 (25%)	20 (38.4%)	36 (40.9%)
Male gender	24 (50%)	17 (32.6%)	45 (51.1%)
Surgical intervention	0 (0%)	3 (3%)	23 (26.13%)
Respiratory support	0 (0%)	0 (0%)	53 (60.22%)
DOH	Discharged at the same day	5.5 (1-35)	24 (3-127)

Values are presented as number (%). *P*: probability value; GA: gestational age (weeks); LBW: low birth weight; VLBW: very low birth weight; DOH: duration of hospitalization; Sig: significance; NS: not significant; HS: highly significant.

**Table 2:** Comparative statistical analysis between the group of sepsis and each of the control groups separately.

	Healthy controls	Diseased controls	Sepsis group	*P*_1_	*P*_2_
	Median (IQR)	Median (IQR)	Median (IQR)	Between healthy controls and sepsis patients	Between diseased controls and sepsis patients
Hb	17 (13.75-18.85)	12.85 (10.325-17.8)	12.4 (10.7-14.7)	<0.001	0.259
TLC	14.3 (12-17.25)	12.7 (9.05-17)	13.3 (8.45-19.1)	0.157	0.648
ANC	5.1 (3.4-11)	4 (2.7-9.6025)	6.5 (4.4-11.8)	0.205	0.007
ALC	5.6 (4.1-7.5)	5.7 (5-6.85)	4 (2.55-6.9)	0.008	0.001
AMC	1.4 (0.9-2)	1 (0.75-1.5)	1.2 (0.7-2)	0.173	0.299
PLT	213 (145-292.25)	348.5 (226.25-408.5)	203 (98.25-307.75)	0.359	<0.001
hs-CRP	5 (5-6)	5 (5-5)	12 (6-39.25)	<0.001	<0.001
nCD11b%	96.95 (95.525-98.1)	97.35 (95.5-98.5)	99.05 (97.1-99.7)	0.446	0.345
nCD11b MFI	5.555 (4.4575-6.22)	5.905 (4.625-6.4525)	5.09 (3.7525-7)	0.619	0.262
mCD14%	84.15 (80.075-89.05)	85.7 (79.1-90.9)	82.7 (58.88-90.7)	0.435	0.11
mCD14 MFI	7.8 (6-8.23)	10.015 (7.7725-11.2)	10.8 (9.315-13.2)	<0.001	0.157

Values are presented as median (IQR). Hb: hemoglobin (g/dL); TLC: total leukocyte count (×10^9^/L); ANC: absolute neutrophil count (×10^9^/L); ALC: absolute lymphocyte count (×10^9^/L); AMC: absolute monocyte count (×10^9^/L); PLT: platelet (×10^9^/L); hs-CRP: highly sensitive CRP (mg/L); nCD11b%: neutrophil CD11b%; nCD11b MFI: nCD11b mean fluorescence intensity; mCD14%: monocyte CD14%; mCD14 MFI: mCD14 mean fluorescence intensity; *P*: a probability value.

**Table 3:** Comparison between documented sepsis (group 2a) and clinical sepsis (group 2b).

	Documented sepsis group	Clinical sepsis group	*Z*	*P*
	Median (IQR)	Median (IQR)		
Hb	10.8 (9.67-12.6)	13.8 (12.2-15.8)	-3.622	<0.001
TLC	13.7 (10.25-16.25)	14.2 (8.4-20)	-0.616	0.538
ANC	8.6 (8.6-5.93)	6.4 (4.6-12.25)	-0.204	0.838
ALC	4.1 (2.6-7)	4.45 (3-8.25)	-0.684	0.494
AMC	1.1 (0.6-2.1)	1.45 (0.73-2.08)	-0.942	0.346
PLT	218.5 (157.75–318)	243 (134-319.5)	-0.152	0.879
hs-CRP	24 (12–85.5)	10 (5-24)	-3.536	<0.001
nCD11b%	99.05 (96.775-99.8)	99.05 (97.1-99.7)	-0.419	0.675
nCD11b MFI	4.44 (3.01-6.3375)	5.31 (4.33- 7.21)	-1.926	0.054
mCD14%	84.9 (57.675-90.7)	82.55 (58.88-90.7)	-0.252	0.801
mCD14 MFI	9.88 (5.92- 11.5)	12.15 (10.3- 16.3)	-3.354	0.001

**Table 4:** Comparison between severe sepsis/septic shock patients and patients with nonsevere sepsis.

	Severe sepsis/septic shock group	Nonsevere sepsis patients	*Z*	*P*
	Median (IQR)	Median (IQR)		
Hb	11.25 (10.28-12.03)	13.3 (10.7- 14.9)	-2.89	<0.001
TLC	9.35 (7.07-13.03)	13.9 (8.9-19.4)	-2.203	0.03
ANC	4.85 (2.26-9.32)	7 (4.9-11.95)	-1.963	0.267
ALC	3.32 (1.25-4.75)	4.2 (2.95-7.15)	-2.333	<0.001
AMC	1.1 (0.4–2)	1.3 (0.7-2.1)	1.28	0.012
PLT	116 (50–195)	234 (158-319)	-3.264	<0.001
hs-CRP	23.9 (9.65-95.9)	12.1 (6.01–37.02)	-2.368	0.018
nCD11b%	98.65 (96.475-99.775)	99.15 (97.1-99.7)	-1.006	0.314
nCD11b MFI	3.6 (2.79-5.4725)	5.53 (4.3025-7.21)	-3.322	0.001
mCD14%	74.24 (50.74-88.5)	83.3 (58.88-90.95)	-1.131	0.258
mCD14 MFI	7.73 (5.07-11.05)	11.1 (9.97-13.9)	-3.181	0.001

**Table 5:** Diagnostic performance of the studied parameters and their combinations arranged in ascending order in terms of their efficacy values.

Parameter	Cutoff value	TN	FP	TP	FN	% specificity	% sensitivity	%NPV	%PPV	%eff.
Hb	8.8	10	87	81	4	10.3	95.3	71.4	48.2	50.0
PLT	524	91	3	6	78	96.8	7.1	53.8	66.7	54.5
mCD14%	93%	87	4	8	72	95.6	10.0	54.7	66.7	55.6
TLC	18.6	83	14	23	62	85.6	27.1	57.2	62.2	58.2
AMC	1.95	73	17	22	49	81.1	31.0	59.8	56.4	59.0
ALC	8.8	88	2	9	61	97.8	12.9	59.1	81.8	60.6
nCD11b MFI	7.73	98	2	16	66	98.0	19.5	59.8	88.9	62.6
ANC	4.4	43	40	59	20	51.8	74.7	68.3	59.6	63.0
mCD14 MFI	9.36	64	25	43	14	71.9	75.4	82.1	63.2	73.3
nCD11b%	99%	94	6	41	41	94.0	50.0	69.6	87.2	74.2
hs-CRP	6	94	6	59	25	94.0	70.2	79.0	90.8	83.2
nCD11b% & hs-CRP	nCD11b% 95.6 & CRP at 6 mg/L	96	4	79	0	96.0	100.0	100.0	95.2	97.8

ANC: absolute neutrophil count (×10^9^/L); Hb: hemoglobin (g/dL); hs-CRP: highly sensitive CRP (mg/L); nCD11b%: neutrophil CD11b%; nCD11b MFI: nCD11b mean fluorescence intensity; mCD14%: monocyte CD14%; mCD14 MFI: mCD14 mean fluorescence intensity; eff.: efficacy; NPV: negative predictive value; PPV: positive predicted value; TP: true positive; TN: true negative; FP: false positive; FN: false negative.

**Table 6:** AUC values for the studied sepsis biomarkers.

Area under the curve	Area	SE	*P*	95% CI	
				Lower	Upper
PLT	0.370	0.052	0.014	0.268	0.473
mCD14%	0.549	0.055	0.349	0.443	0.656
ANC	0.595	0.052	0.071	0.494	0.696
mCD14.MFI	0.703	0.05	<0.001	0.604	0.801
nCD11b%	0.798	0.043	<0.001	0.714	0.883
hs-CRP	0.880	0.038	<0.001	0.805	0.955

**Table 7:** Multiregression analysis.

Multiregression analysis						
Dependent variable: sepsis						
Item	Reg. coef.	*t*	*P*	Sig.	*F*-ratio	*P*
Model 1						
(Constant)	187.536	1.895	0.061	NS		
TLC	-7.074	-0.955	0.342	NS		
ANC	11.332	1.359	0.177	NS		
ALC	-4.315	-0.486	0.628	NS		
AMC	12.428	0.508	0.612	NS		
PLT	-0.144	-1.565	0.12	NS		
nCD11b MFI	-1.975	-0.228	0.82	NS		
mCD14%	-1.245	-1.208	0.23	NS		
mCD14 MFI	13.558	3.543	0.001	HS		
Model 2						
(Constant)	-104.664	-0.784	0.434	NS		
hs-CRP	29.047	6.683	<0.001	HS		
nCD11b%	2.243	1.643	0.102	NS	22.4	<0.001

Hb: hemoglobin (g/dL); TLC: total leukocyte count (×10^9^/L); ANC: absolute neutrophil count (×10^9^/L); ALC: absolute lymphocyte count (×10^9^/L); AMC: absolute monocyte count (×10^9^/L); PLT: platelet (×10^9^/L); hs-CRP: highly sensitive CRP (mg/L); nCD11b%: neutrophil CD11b%; nCD11b MFI: nCD11b mean fluorescence intensity; mCD14%: monocyte CD14%; mCD14 MFI: mCD14 mean fluorescence intensity; *P*: probability value; *Z*^●^: Wilcoxon's rank sum test.

**Table 8:** Delta change percentage for both follow-up groups.

Biomarker	Improved sepsis group	Nonimproved sepsis group	*Z*	*P*
	Median (IQR)	Median (IQR)		
nCD11b%	-0.301 (-5.572-1.131)	0.506 (-2.766-1.945)	-0.567	0.571
nCD11b MFI	-22.475 (-40.638-8.491)	9.663 (-44.489-40.668)	-0.397	0.692
mCD14%	3.515 (-3.297-13.408)	-27.283 (-45.384-6.555)	-1.756	0.079
mCD14 MFI	-1.415 (-29.847-4.088)	-39.515 (-60.331-24.143)	-0.736	0.462

**Table 9:** The predictive validity results for the studied sepsis markers arranged in ascending order in terms of their sensitivities.

	Cutoff	TN	FP	TP	FN	Specificity (%)	Sensitivity (%)	NPV (%)	PPV (%)	Eff. (%)	AUC
nCD11b%	98.5	29	20	11	11	59.2	50.0	72.5	35.5	56.3	0.504
hs-CRP	1.4	40	39	20	19	50.6	51.3	67.8	33.9	50.8	0.456
mCD14%	75.3	35	13	12	10	72.9	54.5	77.8	48.0	67.1	0.594
nCD11b MFI	4.64	34	15	13	9	69.4	59.1	79.1	46.4	66.2	0.620
ANC	6.2	40	31	22	15	56.3	59.5	72.7	41.5	57.4	0.383
mCD14 MFI	9.97	26	11	10	5	70.3	66.7	83.9	47.6	69.2	0.673
mCD14 MFI & mCD14%	CD14 MFI at 9.97 & CD14% at 44.7%	36	1	10	5	97.3	66.7	87.8	90.9	88.5	0.846

Eff.: efficacy; NPV: negative predictive value; PPV: positive predicted value; TP: true positive; TN: true negative; FP: false positive; FN: false negative.

## Data Availability

The authors declare that the data underlying the findings of this research are publicly available.

## Supplementary Materials

Supplementary Materials Supplementary Table 1: statistical comparison between the control group and the documented sepsis patients; significant differences were observed between both groups by each of the following sepsis parameters: hs-CRP, PLT, Hb, ALC, and nCD11b MFI. Supplementary Figure S1: diagnostic validity results of studied sepsis markers; the highest sensitivity, specificity, and efficacy achieved by the combined measurement of nCD11b% and hs-CRP. Supplementary Table 2: the correlations between nCD11b%, nCD11b MFI, mCD14%, mCD14 MFI, and the conventional sepsis parameters; both nCD11b and mCD14 failed to show a significant correlation with the other studied parameters except for a positive correlation between both biomarkers of MFI (i.e., nCD11b MFI and mCD14 MFI). A nonsignificant correlation was documented between hs-CRP and any of nCD11b%, nCD11b MFI, mCD14%, and mCD14 MFI. Supplementary Table 3: a comparison between the first baseline and the 2^nd^ monitoring evaluations regarding the improved sepsis group; significantly different values were achieved by each of the following sepsis parameters: hs-CRP, PLT, nCD11b MFI, ANC, and Hb. Supplementary Table 4: a comparison between the first baseline and the 2^nd^ monitoring evaluations regarding the nonimproved sepsis patients' group; all studied sepsis parameters show a nonsignificant difference (>0.05) between both evaluations.
