# Supplementary material for: Diagnostic, Prognostic, Predictive, and Monitoring Role of Neutrophil CD11b and Monocyte CD14 in Neonatal Sepsis
Source: Dis Markers. 2021 Oct 14;2021:4537760. doi: 10.1155/2021/4537760 (PMC8531823; doi:10.1155/2021/4537760)
Supplement: Supplementary Materials — Supplementary Table 1: statistical comparison between the control group and the documented sepsis patients; significant differences were observed between both groups by each of the following sepsis parameters: hs-CRP, PLT, Hb, ALC, and nCD11b MFI. Supplementary Figure S1: diagnostic validity results of studied sepsis markers; the highest sensitivity, specificity, and efficacy achieved by the combined measurement of nCD11b% and hs-CRP. Supplementary Table 2: the correlations between nCD11b%, nCD11b MFI, mCD14%, mCD14 MFI, and the conventional sepsis parameters; both nCD11b and mCD14 failed to show a significant correlation with the other studied parameters except for a positive correlation between both biomarkers of MFI (i.e., nCD11b MFI and mCD14 MFI). A nonsignificant correlation was documented between hs-CRP and any of nCD11b%, nCD11b MFI, mCD14%, and mCD14 MFI. Supplementary Table 3: a comparison between the first baseline and the 2nd monitoring evaluations regarding the improved sepsis group; significantly different values were achieved by each of the following sepsis parameters: hs-CRP, PLT, nCD11b MFI, ANC, and Hb. Supplementary Table 4: a comparison between the first baseline and the 2nd monitoring evaluations regarding the nonimproved sepsis patients' group; all studied sepsis parameters show a nonsignificant difference (>0.05) between both evaluations. [file 4537760.f1.docx]

**Table (S1):** Comparison between control group and documented sepsis patients.

|  | **Control group** | **Documented sepsis group** | **Z** | **p** |
| --- | --- | --- | --- | --- |
| **Hb** | 15.1(11.4- 18.35) | 11.15(9.825-12.075) | -4.971 | <0.001 |
| **TLC** | 13.7(9.9-17.2) | 12.4(8.45-14.9) | -1.806 | 0.071 |
| **ANC** | 4.4(2.9- 10.4) | 6.75(3.3- 10.25) | -0.98 | 0.327 |
| **ALC** | 5.7(4.425-7.25) | 3.55(2.15-5.3) | -4.303 | <0.001 |
| **AMC** | 1.25(0.8-1.9) | 1.1(0.46- 2) | -1.082 | 0.279 |
| **PLT** | 269(181.75-359.5) | 167(90- 250) | -3.826 | <0.001 |
| **Hs-CRP** | 0.5)0.5- 0.5) | 2.3(1.125- 4.525) | -8.427 | <0.001 |
| **nCD11b %** | 97.1(95.5- 98.475) | 99.05(96.775-99.8) | -4.348 | 0.345 |
| **nCD11b.MFI** | 5.61(4.6- 6.4) | 4.44(3.01- 6.3375) | -1.995 | 0.046 |
| **mCD14 %** | 85.2(79.1- 90.7) | 84.9(57.675-90.7) | -1.027 | 0.304 |
| **mCD14.MFI** | 8.1(7.08- 10.45) | 9.88(5.92- 11.5) | -1.407 | 0.16 |

Values are presented as Median and (IQR)

Hb (hemoglobin) g/dl

TLC (total leukocytic count) (X10^9^ / L)

ANC: Absolute neutrophil count (X10^9^ / L)

ALC: Absolute lymphocyte count (X10^9^ / L)

AMC; Absolute monocyte count (X10^9^ / L)

PLT (Platelet) (X10^9^ / L)

Hs-CRP (highly sensitive CRP) mg/L

nCD11b% (neutrophil CD11b%)

nCD11b MFI (nCD11b mean fluorescence intensity)

mCD14% (monocyte CD14%)

mCD14 MFI (mCD14 mean fluorescence intensity)

P, probability value

Z ^●^ = Wilcoxon's Rank Sum Test

Sig, significance

NS, not-significant.

HS, highly significance

**Figure (1s):** diagnostic validity results of studied sepsis markers, the highest sensitivity, specificity, and efficacy achieved by the combined measurement of (nCD11b% and hs-CRP). Despite that mCD14, MFI recorded the highest sensitivity could be achieved by a univariant marker in the study, but unsatisfactory specificity and efficacy were documented by this marker. nCD11b% achieved the highest specificity as a sole marker but it is associated with poor sensitivity, while Hs-CRP is associated with the highest univariant marker efficacy.

|  | **nCD11b %** | | **nCD11b.MFI** | | **mCD14 %** | | **mCD14.MFI** | | |
| --- | --- | --- | --- | --- | --- | --- | --- | --- | --- |
|  | **rs** | **P** | **rs** | **P** | **rs** | **P** | **rs** | **P** | |
| **GA** | -0.039 | 0.733 | 0.067 | 0.559 | 0.029 | 0.805 | 0.235 | | 0.087 |
| **BW** | 0.007 | 0.949 | -0.099 | 0.392 | 0.162 | 0.164 | 0.079 | | 0.571 |
| **Hb** | -0.181 | 0.108 | 0.049 | 0.663 | -0.053 | 0.644 | 0.183 | | 0.18 |
| **TLC** | -0.104 | 0.36 | 0.062 | 0.582 | 0.032 | 0.784 | 0.105 | | 0.446 |
| **ANC** | -0.019 | 0.872 | -0.091 | 0.439 | 0.098 | 0.41 | 0.09 | | 0.526 |
| **ALC** | -0.125 | 0.323 | 0.244 | 0.05 | -0.038 | 0.761 | 0.115 | | 0.433 |
| **AMC** | -0.169 | 0.174 | 0.062 | 0.619 | 0.065 | 0.6 | 0.077 | | 0.601 |
| **PLT** | 0.085 | 0.453 | 0.244 | 0.03 | 0.037 | 0.751 | 0.186 | | 0.174 |
| **hs-CRP** | -0.027 | 0.811 | -0.187 | 0.099 | 0.056 | 0.627 | -0.187 | | 0.176 |
| **nCD11b %** |  |  | 0.132 | 0.238 | 0.481 | <0.001 | -0.074 | | 0.586 |
| **nCD11b.MFI** |  |  |  |  | -0.086 | 0.462 | 0.299 | | 0.024 |
| **mCD14 %** |  |  |  |  |  |  | 0.179 | | 0.182 |

**Table (S2):** the correlations between nCD11b%, nCD11b MFI, mCD14%, mCD14 MFI and the conventional sepsis parameters.

GA: Gestational Age

BW: birth weight

Hb (hemoglobin) g/dl

TLC (total leukocytic count) (X10^9^ / L)

ANC: Absolute neutrophil count (X10^9^ / L)

ALC: Absolute lymphocyte count (X10^9^ / L)

AMC; Absolute monocyte count (X10^9^ / L)

PLT (Platelet) (X10^9^ / L)

Hs-CRP (highly sensitive CRP) mg/L

nCD11b% (neutrophil CD11b%)

nCD11b MFI (nCD11b mean fluorescence intensity)

mCD14% (monocyte CD14%)

mCD14 MFI (mCD14 mean fluorescence intensity)

P, probability value

**Table (S3):** Comparison between baseline and follow up evaluations for improved sepsis groups.

| **Biomarkers**  **(Improved sepsis group)** | **Initial level** | **Final level** |  |  |
| --- | --- | --- | --- | --- |
|  | **Median (IQR)** | **Median (IQR)** | **Z** | **p** |
| **Hb** | 12.7 (10.4 – 14.8) | 11.6(10.2 – 13) | -2.212 | 0.027 |
| **TLC** | 13.5 (9 – 18.2) | 13 (10- 15.3) | -1.535 | 0.125 |
| **ANC** | 6.08(4.55- 11.15) | 5.57 (3.725-7.075) | -2.783 | 0.005 |
| **ALC** | 4.1 (2.55-6.95) | 4.6(3.5-6.05) | -0.350 | 0.726 |
| **AMC** | 1.35(0.7-2.2) | 0.94 (0.5-1.2) | -1.859 | 0.063 |
| **PLT** | 197 (106 -319) | 283 (200 – 447) | -3.459 | 0.001 |
| **hs-CRP** | 23 (8 – 48) | 6 (5- 23.75) | -3.968 | <0.001 |
| **nCD11b %** | 98.7(98.3-99.6) | 98.9(94.1-99.8) | -0.801 | 0.423 |
| **nCD11b.MFI** | 6.17(5.3-7.21) | 5.39(3.45-5.53) | -2.045 | 0.041 |
| **mCD14 %** | 88.2(49.8- 90.9) | 88(75.9- 91.3) | -1.274 | 0.203 |
| **mCD14.MFI** | 11.45(8.8875-13.225) | 9.01(7.83-12.25) | -1.120 | 0.263 |

Initial level: first evaluation

final level: the 2^nd^ evaluation

Hb (hemoglobin) g/dl

TLC (total leukocytic count) (X10^9^ / L)

ANC: Absolute neutrophil count (X10^9^ / L)

ALC: Absolute lymphocyte count (X10^9^ / L)

AMC; Absolute monocyte count (X10^9^ / L)

PLT (Platelet) (X10^9^ / L)

Hs-CRP (highly sensitive CRP) mg/L

nCD11b% (neutrophil CD11b%)

nCD11b MFI (nCD11b mean fluorescence intensity)

mCD14% (monocyte CD14%)

mCD14 MFI (mCD14 mean fluorescence intensity)

P, probability value

Z ^●^ = Wilcoxon's Rank Sum Test

Sig, significance

NS, not-significant.

HS, highly significance

**Table (S4):** Comparison between the baseline and follow up evaluations for the non-improved sepsis group.

| **Biomarkers**  **(non-improved sepsis group)** | **Initial level** | **Final level** |  |  |
| --- | --- | --- | --- | --- |
|  | **Median (IQR)** | **Median (IQR)** | **Z** | **p** |
| **Hb** | 12.4(10.85- 14.025) | 11.15(10.3 – 13.8) | -1.349 | 0.177 |
| **TLC** | 14.7(9.475-18.975) | 14.3(9.125- 23.4) | -0.411 | 0.681 |
| **ANC** | 6.5(3.9-9.1) | 9.2 (4.7 – 12.5) | -1.412 | 0.158 |
| **ALC** | 4.3(3.115-5.11) | 2.685(0.8175- 3.39) | -1.782 | 0.075 |
| **AMC** | 1.8 (1.1 – 2.6) | 1 (0.45 – 2.8) | -1.014 | 0.311 |
| **PLT** | 209(120 – 288) | 158(88- 234) | -1.046 | 0.295 |
| **hs-CRP** | 10.5(1.25 – 22) | 24(7.5 – 79.5) | -1.735 | 0.083 |
| **nCD11b %** | 98.8(97.85-99.4) | 99.3(96.65- 99.75) | -0.405 | 0.686 |
| **nCD11b.MFI** | 7(4.35-9.17) | 5.53(4.045- 9.045) | -0.405 | 0.686 |
| **mCD14 %** | 89.5(80.05-91.9) | 66.9(46.55-89.65) | -1.483 | 0.138 |
| **mCD14.MFI** | 10.3(9.705- 12.2) | 6.23(4.29- 13.085) | -0.674 | 0.5 |

Initial level: first evaluation

final level: the 2^nd^ evaluation

Hb (hemoglobin) g/dl

TLC (total leukocytic count) (X10^9^ / L)

ANC: Absolute neutrophil count (X10^9^ / L)

ALC: Absolute lymphocyte count (X10^9^ / L)

AMC; Absolute monocyte count (X10^9^ / L)

PLT (Platelet) (X10^9^ / L)

Hs-CRP (highly sensitive CRP) mg/L

nCD11b% (neutrophil CD11b%)

nCD11b MFI (nCD11b mean fluorescence intensity)

mCD14% (monocyte CD14%)

mCD14 MFI (mCD14 mean fluorescence intensity)

P, probability value

Z ^●^ = Wilcoxon's Rank Sum Test

NS, not-significant.
